# YIM-P58. Macrophage activation syndrome: the role of infectious triggers

**DOI:** 10.1186/1546-0096-12-S1-Y5

**Published:** 2014-09-17

**Authors:** Maria Maddalena D'Errico, Federica Cuoco, Carlo Biancardi, Marta Torcoletti, Giovanni Filocamo, Fabrizia Corona

**Affiliations:** 1 UOS Reumatologia Pediatrica, Fondazione IRCCS Ca' Granda, Ospedale Maggiore Policlinico, Milano, Italy

## Introduction

Macrophage activation syndrome (MAS) is a potentially fatal complication of childhood rheumatic diseases (RD), due to excessive activation and proliferation of macrophages. It belongs to secondary forms of Hemophagocytic lymphohistiocytosis (HLH), including HLH-infection associated forms (HLH-IA).

## Objectives

The aim of our study is to assess the prevalence of clinical and laboratory features, possible triggers and outcomes of MAS.

## Methods

We retrospectively evaluated a cohort of 12 patients with MAS and HLH-IA, observed at the U.O.S. Pediatric Rheumatology, from 2005 to 2014. Here we report: sex, ethnicity, RD, age at the onset of the rheumatic disease, age at the onset of MAS, duration of the disease pre-MAS; clinical and laboratory features; results of the bone marrow biopsy if performed; trigger factors, treatments and outcomes.

## Results

We identified 12 patients: 9 MAS and 3 HLH-IA; 9 females and 3 males; 10 Caucasians, 1 Egyptian and 1 Latin American. The mean age at diagnosis of MAS was 9.25 years. 9 children had RD: 6 systemic juvenile idiopathic arthritis, 1 dermatomyositis, 1 autoinflammatory disease and 1 systemic lupus erythematosus. In 3 cases MAS occurred during the first presentation of the RD. In all cases, RD status was active during MAS. The mean age at onset of RD was 7.8 years. HLA-IA and MAS could occur at the onset of the RD with an interval of 22 days or later in the course of RD with an interval of 1105 days on average. Organ involvement was: hepatomegaly 12, splenomegaly 7, lymphoadenopathy 5, bleeding 2, central nervous system 6, heart 8, lung 4, kidney 5 and gallbladder 2.

Table 1 summarizes patients' characteristics, the clinical and laboratory features and number of cases.

**Table 1:** 

Persistent fever ≥38°C	12	Decreased platelet count	12
** Hyperferritinemia (≥1000 ng/ml) **	12	** Decreased erythrocyte sedimentation rate **	12

** Increased liver enzyme levels **	11	** Decreased leukocyte count **	11

** Hypofibrinogenemia **	10	** Hypertriglyceridemia **	9

** Hemophagocytosis in bone marrow biopsy **	6/8		

In 11 cases the possible trigger was an infectious episode. We identified 5 bacterial infections (Chlamydia pneumoniae, Mycoplasma pneumoniae, Clostridium difficilis, group A beta-hemolytic Streptococcus, Staphylococcus aureus), 2 viral infections Chickenpox virus, Epstein-Barr virus and one multiple protozoal infection (Entamoeba histolytica, Endolimax nana). In 4 cases there were signs suggestive of infection, including lower respiratory tract infection, skin infection, urinary tract infection, gastroenteritis, but it was not possible to identify a pathogen.

All patients were treated with methylprednisolone and cyclosporine. 9 received transfusions of fresh frozen plasma, 7 blood transfusions and 4 intravenous immunoglobulins. Regarding the outcomes, 1 patient died. 3 had sequelae: seizures in 1, 1 spastic quadriplegia and 1 flaccid paralysis of half body, with a motor type polyneuropathy.

## Conclusion

In our cases, RD was in active phase at the time of diagnosis of MAS, regardless of the time elapsed since the first diagnosis. The infection appears to have a triggering role in the MAS, including Entamoeba hystolitica and Endolimax nana, as far we are aware, are not reported in the literature.

## Disclosure of interest

None declared.

